# Supplementary material for: Comparative Mutagenic Effectiveness and Efficiency of Gamma Rays and Sodium Azide in Inducing Chlorophyll and Morphological Mutants of Cowpea
Source: Plants (Basel). 2022 May 16;11(10):1322. doi: 10.3390/plants11101322 (PMC9144755; doi:10.3390/plants11101322)

Figure S1. Region-wise production share of Cowpeas (Average 2005–2016).

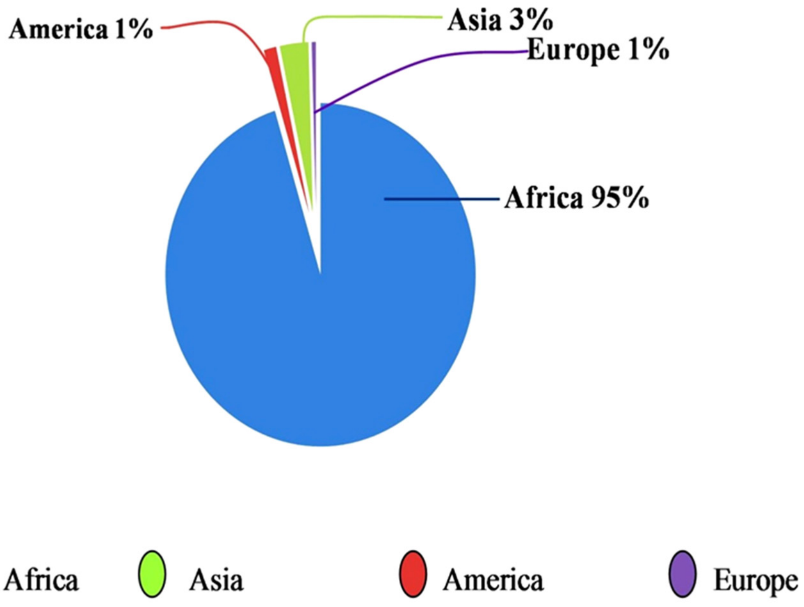

Figure S2. Field layout and pattern of seed sowing in a randomized complete block design (RCBD) for each cultivar (Source: Raina et al., 2020. http://creativecommons.org/licenses/by/4.0/).

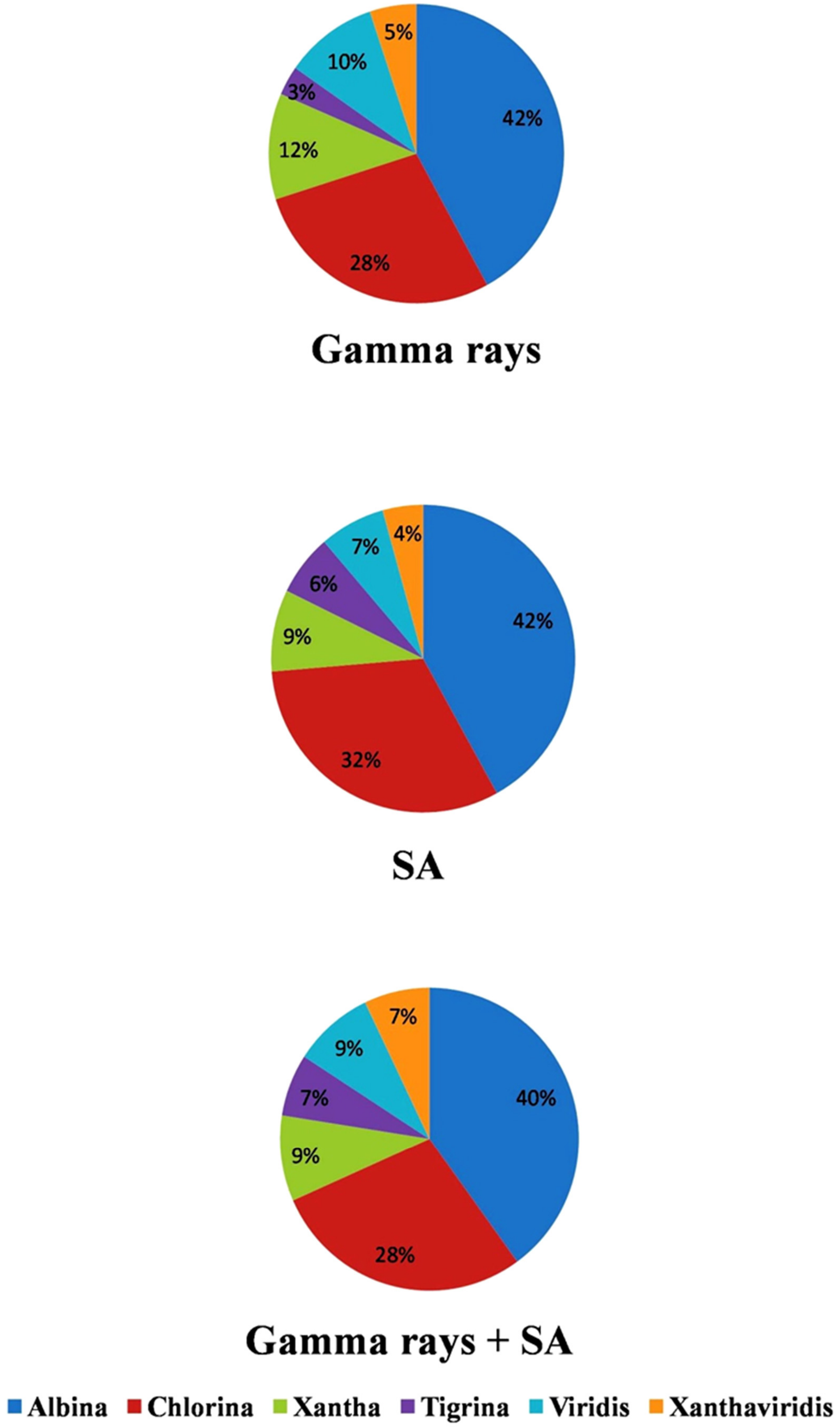

Figure S3. Comparative frequency and spectrum of chlorophyll mutations based on the pooled values of two varieties.

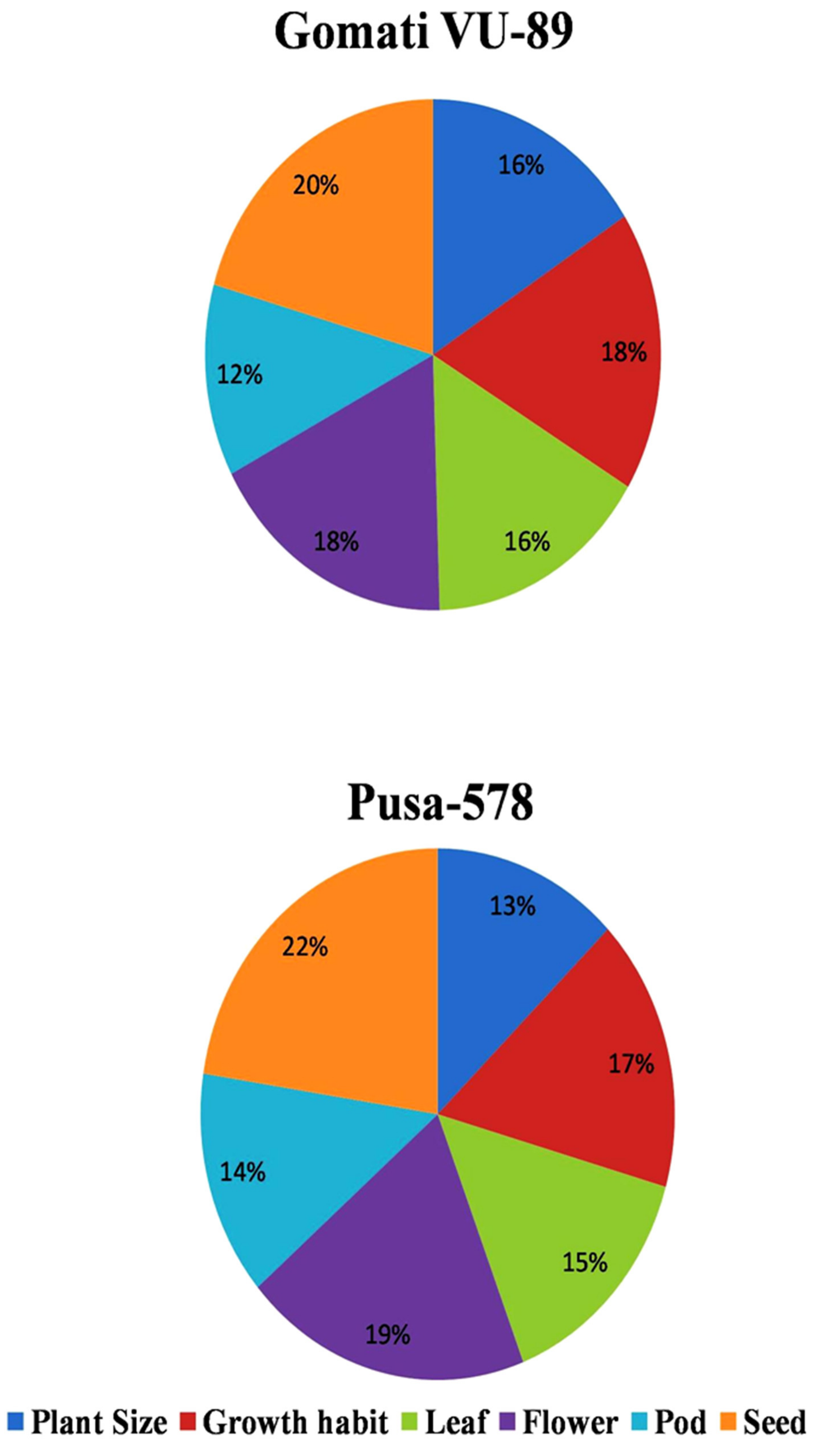

Figure S4. Comparative frequency and spectrum of induced morphological mutations in cowpea are based on the pooled values of two varieties.

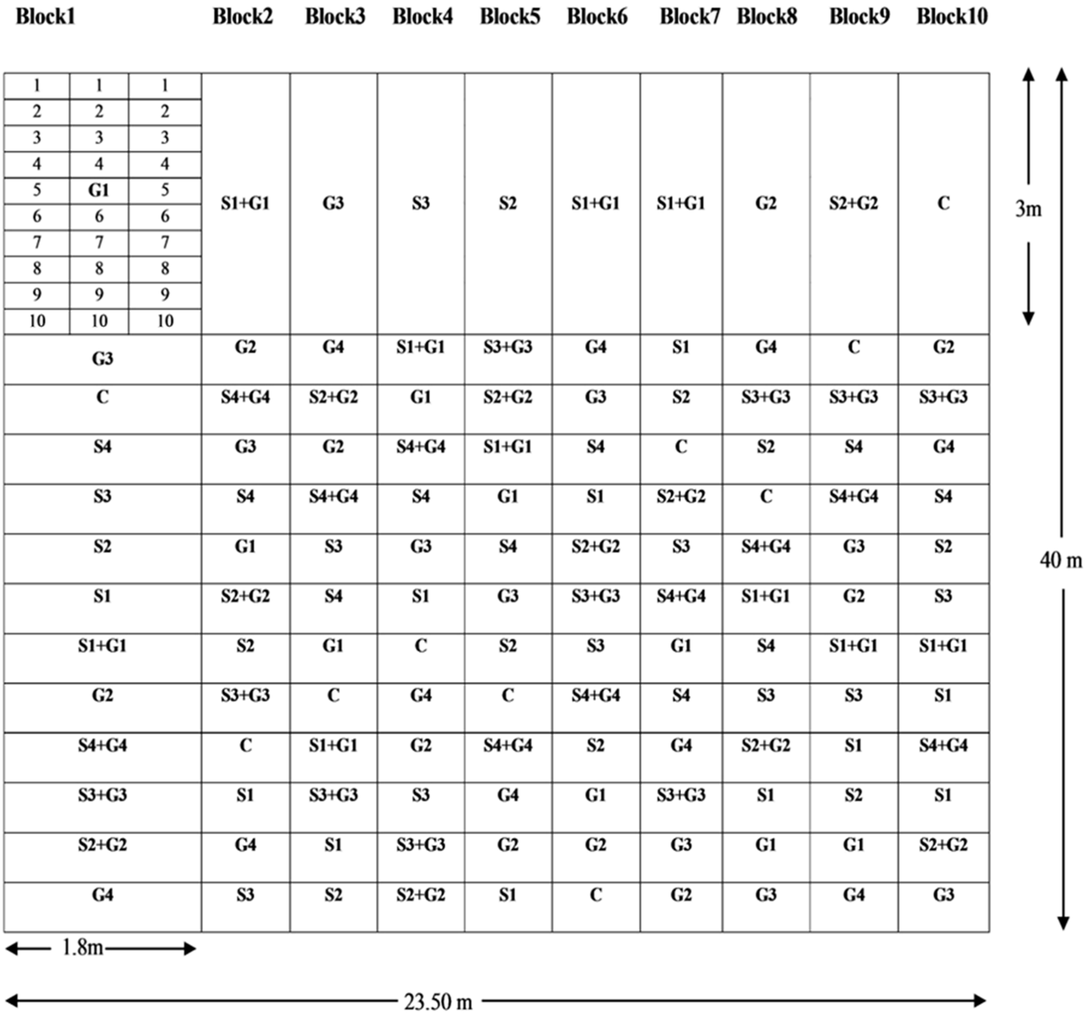

Supplement: Supplementary file 1 [file plants-11-01322-s001.zip › Figures S1-S4.pdf]
