# Supplementary material for: Comparative Mutagenic Effectiveness and Efficiency of Gamma Rays and Sodium Azide in Inducing Chlorophyll and Morphological Mutants of Cowpea
Source: Plants (Basel). 2022 May 16;11(10):1322. doi: 10.3390/plants11101322 (PMC9144755; doi:10.3390/plants11101322)
Supplement: Supplementary file 1 [file plants-11-01322-s001.zip › Table S1.pdf]

Table S1 Details of the varieties used in the present study.

	**Pedigree**	**Year / Place of release**	**Distinguishing characters**
**var. Gomati VU-89**	Selection from local germplasm at Kanke	1974/ RAU, Kanke, Ranchi	Maturity 150-155 days; Avg. yield 25-30 Q/ha; Resistant to yellow mosaic virus; 13.00 g/100 seeds; Long pods; Avg. 60 pods/plant; small seed size
**var. Pusa-578**	Selection from cv. EC170578	2005/ IARI, New Delhi	Maturity 155-160 days; Avg. yield 5 Q/ha; Resistant to yellow mosaic virus; 21.00 g/100 seeds; Short pods; Avg. 40 pods/plant; Large seed size
