# Supplementary material for: Comparative Mutagenic Effectiveness and Efficiency of Gamma Rays and Sodium Azide in Inducing Chlorophyll and Morphological Mutants of Cowpea
Source: Plants (Basel). 2022 May 16;11(10):1322. doi: 10.3390/plants11101322 (PMC9144755; doi:10.3390/plants11101322)
Supplement: Supplementary file 1 [file plants-11-01322-s001.zip › Table S2.pdf]

Table S2: Description of seed germination and plant survival in each generation from M_1 to M_2 in the varieties Gomati VU-89 and Pusa-578.

**Mutagens**	**Gomati VU-89**						
	**M_1**			**M_2**			
	**Seed Treated**	**Seeds Germinated**	**Fertile Plants**	**M_2 Lines**	**Total M_2 Seeds**	**Seeds Germinated**	**Fertile Plants**
C*	0	278	276	276	2760	2576	2378
G1	300	261	260	260	2600	2297	2013
G2	300	255	254	254	2540	2184	1864
G3	300	245	243	243	2430	2009	1634
G4	300	240	238	238	2380	1920	1542
S1	300	260	259	259	2590	2262	1975
S2	300	254	252	252	2520	2167	1835
S3	300	246	243	243	2430	2025	1647
S4	300	238	233	233	2330	1895	1478
G1S1	300	257	255	255	2550	2219	1893
G2S2	300	245	243	243	2430	2033	1660
G3S3	300	235	230	230	2300	1848	1429
G4S4	300	228	225	225	2250	1748	1316
T	**3600**	**2964**	**2935**	**2935**	**29350**	**24607**	**20287**
**Mutagens**	**Pusa-578**						
	**M_1**			**M_2**			
C*	0	272	270	270	2700	2484	2269
G1	300	251	250	250	2500	2125	1792
G2	300	245	244	244	2440	2033	1660
G3	300	230	238	238	2380	1944	1562
G4	300	220	233	233	2330	1872	1466
S1	300	250	258	258	2580	2279	1983
S2	300	244	242	242	2420	2033	1653
S3	300	229	233	233	2330	1856	1460
S4	300	219	226	226	2260	1725	1317
G1S1	300	242	245	245	2450	2050	1688
G2S2	300	225	232	232	2320	1872	1454
G3S3	300	210	224	224	2240	1732	1311
G4S4	300	205	202	202	2020	1522	1116
T	**3600**	**2770**	**2827**	**2827**	**28270**	**23043**	**18462**

*For control (C) set 300 seeds per variety were also sown in the same field.
